# Phytochemical Analysis, Antimicrobial and Antioxidant Properties of *Thymus zygis* L. and *Thymus willdenowii* Boiss. Essential Oils

**DOI:** 10.3390/plants11010015

**Published:** 2021-12-22

**Authors:** Fatima zahrae Radi, Mohamed Bouhrim, Hamza Mechchate, Mohammed Al-zahrani, Ashraf Ahmed Qurtam, Abdulmalik M. Aleissa, Aziz Drioiche, Nadia Handaq, Touriya Zair

**Affiliations:** 1 Research Team of Chemistry Bioactive Molecules and the Environment, Laboratoire des Matériaux Innovants et Biothenologie des Resources Naturelles, University Moulay Ismaïl of Meknes-Faculty of Sciences, Meknes 50000, Morocco; fat.radi@edu.umi.ac.ma (F.z.R.); mohamed.bouhrim@gmail.com (M.B.); a.drioiche@edu.umi.ac.ma (A.D.); Hadaq.nadia@umi.ac.ma (N.H.); 2 Laboratory of Inorganic Chemistry, Department of Chemistry, University of Helsinki, P.O. Box 55, FI-00014 Helsinki, Finland; 3 Biology Department, College of Science, Imam Mohammad Ibn Saud Islamic University (IMSIU), Riyadh 11623, Saudi Arabia; MMyAlzahrani@imamu.edu.sa (M.A.-z.); AAQURTAM@imamu.edu.sa (A.A.Q.); 4 King Khaled Eye Specialist Hospital (KKESH), Riyadh 11462, Saudi Arabia; aaleissa@kkesh.med.sa

**Keywords:** *Thymus zygis* L., *Thymus willdenowii* Boiss, volatile compounds, GC-MS analysis, bacteria, fungi, molds, multi-resistant bacteria

## Abstract

Essential oils (EOs) are chemical products produced by odoriferous glands from a variety of plants. These essential oils have many health benefits: antiseptic, anti-inflammatory and antimicrobial activities. So due to these medicinal properties, the present study was designed to analyze essential oils of *Thymus zygis* L. and *Thymus willdenowii* Boiss. for their chemical composition and biological activities. These two thyme species were collected from the region of Ifrane, Middle Atlas of Morocco. The EO was obtained by hydrodistillation, and the yields were 5.25% for *T. zygis* and 3.00% for *T. willdenowii*. The chemical composition of the EOs was analyzed by gas chromatography coupled with mass spectrometry (GC-MS), and the results showed that *T. zygis* EO is dominated by carvacrol (52.5%), *o-*cymene (23.14%), and thymol (9.68%), while the EO of *T. willdenowii* contains germacrene D (16.51%), carvacrol (16.19%), and geranyl acetate (8.35%) as major compounds. The antioxidant activity assessed by Diphenylpicrylhydrazyl (DPPH) and ferric reducing antioxidant power (FRAP) assays revealed that both EOs have excellent antioxidant activities; by DPPH it resulted in IC_50_ = 6.13 ± 0.11 for *T. zygis* and 6.78 ± 0.3 µg/mL for *T. willdenowii*, while the one by FRAP yielded EC_50_ = 2.46 ± 0.01 (*T. zygis*) and 5.17 ± 0.2 (*T. willdenowii*) µg/mL. The antimicrobial activity of the two essential oils was evaluated against six bacterial strains and five fungal strains by the disk diffusion method to determine the Minimum Inhibitory Concentration (MIC), Minimum Bactericidal Concentration (MBC) and Minimum Fungicidal Concentration (MFC). The EOs revealed variable antimicrobial activities against the different tested microbial strains and showed strong antimicrobial activities, even against strains known as multi-resistant to antibiotics (*Acinetobacter baumannii*) at low concentrations (2 µL/mL). *T. zygis* EO showed the most powerful activity against all the studied bacteria, while that of *T. willdenowii* recorded moderate activity when tested against *Shigella dysenteriae* and *Salmonella* Typhi. With inhibition diameters that vary between 75 mm and 84 mm for concentrations of 2 µL/mL up to 12 µL/mL, *S. aureus* was shown to be the most sensitive to *T. zygis* EO. For the antifungal activity test, *T. zygis* EO showed the best inhibition diameters compared to *T. willdenowii* EO. These results showed that *T. zygis* EO has more powerful antioxidant and antimicrobial activities than *T. willdenowii* EO, therefore, we deduce that thyme EOs are excellent antioxidants, they have strong antimicrobial properties, and may in the future represent new sources of natural antiseptics that can be used in pharmaceutical and food industry.

## 1. Introduction

In recent years, there has been increasing interest in natural substances of plant origin with therapeutic potential. This increase has been linked to several factors, including beneficial health effects, in particular with the extracts and products derived from higher plants, which have led to the discovery, and the development of useful therapeutic agents [1,2]. These products are relatively low-toxic, inexpensive, available, and have effects against many pathologies (bacteria, fungus, viruses, parasites, etc.) that pose infection risks to the human body. Let us quote, for example, the essential oils whose actions against bacteria were realized in 1881 by Delacroix [3]. Since then, many essential oils have been recognized as efficient antimicrobial natural products. The activities of many essential oils have been studied during this time, like thyme, lemongrass, cinnamon, and others [4]. Numerous studies have approved their bioactivity in fighting bacteria, fungi, diabetes, oxidative stress, kidney problems, and many others [5,6]. Among the plants known for their therapeutic effects, we note thyme. This plant is commonly used as a spice and has considerable virtues thanks to the progressive discovery of its applications in care and beauty, as well as its uses in culinary practices. Thyme, a wild aromatic plant belonging to the Lamiaceae family, is found mainly in the Mediterranean region, Asia, Southern Europe, and North Africa [7]. Almost 100 species are identified throughout the world [8], and in Morocco, there are 21 species of thyme, 10 of which are endemic to Morocco (*Thymus maroccanus*, *Thymus bleicherianus*, *Thymus atlanticus*, *Thymus satureioides*, *Thymus broussonnetii*, *Thymus leptobotrys*, *Thymus pallidus* subsp. *pallidus*, *Thymus pallidus* subsp. *eriodontus*, *Thymus riatarum*, *Thymus serpyllum*) [9]. The whole plant (thym) is widely used in traditional medicine [10,11]. Its essential oil is widely used in alternative medicine as antiseptic, antispasmodic, antimicrobial, and antioxidant [12,13,14]. Thyme has been used in traditional Moroccan medicine in the treatment of diarrhea, fever, cough, infested zones, and wounds. It was also used as a tonic and stimulant [6,15] and has anti-inflammatory properties after topical application or oral administration [16]. The flowering tops of thyme mainly contain flavonoids (derivatives of apigenol and luteolol), phenol acids (especially caffeic and rosmarinic acids), tannins, resin, and its essential oil is very rich in terpenes, which are responsible for the majority of the pharmacological effects [17]. 

Considering the popular use of the plants from this family in traditional medicine to relieve certain pains and treat certain diseases [6] we have selected two species from the Middle Atlas of Morocco (Region of Ifrane), namely *Thymus zygis* L. and *Thymus willdenowii* Boiss. (commonly called Zaâitra or Azoukeni in Berber) in order to investigate and compare the chemical compositions, antioxidant, and antimicrobial activity against multidrug-resistant bacteria (*Escherichia coli*, *Staphylococcus aureus*, *Acinetobacter baumannii*, *Shigella dysenteriae*, *Salmonella* Typhi, and *Enterobacter cloacae*) fungi (yeasts (*Candida albicans*, *Candida glabrata*, and *Candida* spp.) and molds (*Aspergillus fischeri*, and *Fusarium solani*)) and determine their antioxidant activities using the DPPH and FRAP methods.

## 2. Results and Discussion

### 2.1. Phytochemical Study

#### The Yield of Essential Oils

The results of the essential oils yields obtained by hydrodistillation from samples of *T. zygis* and *T. willdenowii* are given in Table 1. With 5.25%, the *T. zygis* sample provided the highest yield against only 3.00% obtained with *T. willdenowii.* The latter remains higher compared to that obtained by El Idrissi and Idrissi (0.28%) [18] and also when compared to other thyme species in Morocco such as *Thymus bleicherianus* collected in Meknes (Center of Morocco) (1.71%), *Thymus capitatus* collected in Tetouan (North of Morocco) (1.43%) and *Thymus satureioides* collected in Agadir (southwest of Morocco) (0.69%) [19].

The obtained *T. zygis* EO yield is much higher than that harvested in Portugal by Moldão-Martins with 1.2% [20] and 3% yielded by Sotomayor et al. for *T. zygis* ssp. *gracilis* [21,22]. A similar yield (2.3 to 3.6%) was found by Jordan et al. [23]. These differences in yields of essential oils could be explained by several factors, including crop origin, genetic factors, geographical position, soil type, climatic conditions, weather, and extraction apparatus [24,25,26].

### 2.2. Physicochemical Characteristics of the Selected Thyme Essential Oils (Density, Refractive Index, and Brix Degree)

The density, refractive index and Brix degree are qualitative identification characteristics that may be used to evaluate the purity of essential oils. Each substance has its specific refractive index. The purity of a product is determined by how near its refractive index is to the anticipated value. According to Table 2 given below, the density of the essential oils of *T. zygis* (0.92 ± 0.05) was slightly higher than that of the essential oil of *T. willdenowii* (0.91 ± 0.05). The same observation was made on the refractive index and the Brix degree. *T. zygis* had the highest values with 1.50 ± 0.05 and 85.44 ± 0.05%, respectively. The density and refractive index values of *T. zygis* and *T. willdenowii* essential oils extracted by hydrodistillation are comparable to those of standards, indicating that our extracts are of excellent purity. In fact, in accordance with ISO 14715: 2010, the density of essential oils of thymol thyme (*Thymus zygis* (Loefl.) L.) varies between 0.91 and 0.93, and the refractive index varies between 1.494 and 1.50.

### 2.3. Chemical Composition of T. zygis and T. willdenowii Essential Oils 

**Figure 1:**
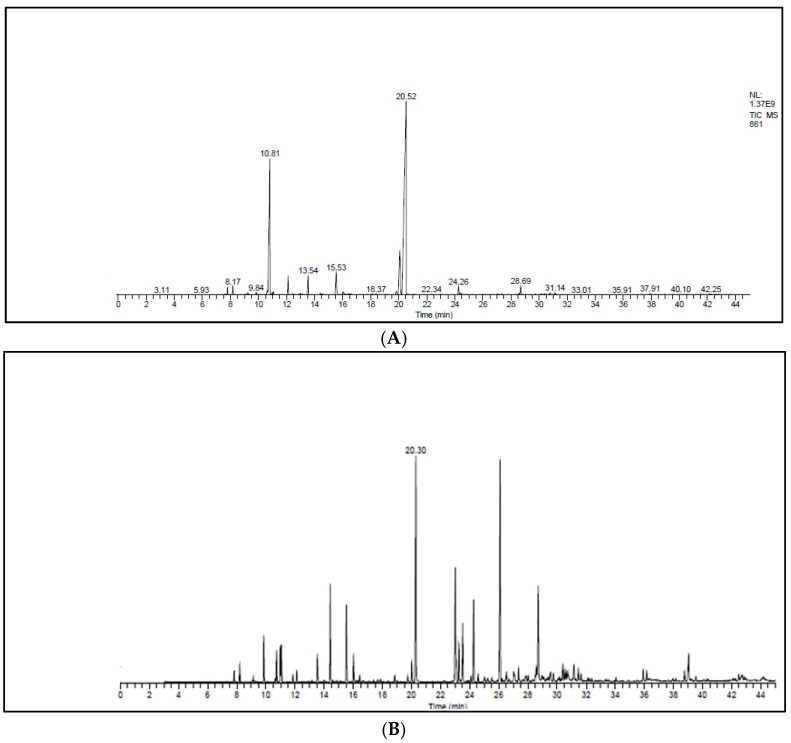
Chromatograms of the EOs. (**A**) *T. zygis*, (**B**) *T. willdenowii*.

**Figure 2:**
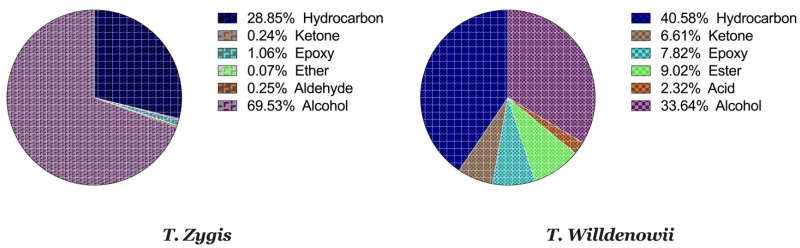
Percentage of chemical families in the thymes EO.

Figure 1A,B and Table 3 represent the chromatograms and the details of the composition of the two EOs with their intensities. A total of 31 compounds were identified in the EO of *T. zygis* (sum of approximately 99.84%), while 33 compounds were identified in that of *T. willdenowii* (sum of approximately 98.69%). The chemical composition of *T. zygis* EO consists mainly of oxygenated monoterpenes (68.7%) and hydrocarbon monoterpenes (27.55%) with carvacrol (52.20%), *o-*cymene (23.14%), and thymol (9.68%) as major compounds, accompanied by other compounds at relatively low levels (borneol (3.30%), linalool (2.40%), γ-terpinene (1.98%) and caryophyllene oxide (1.06%)). Analyzing further the chemical composition of this EO, six families were noted: Alcohols (69.53%), hydrocarbons (28.85%), epoxides (1.06%), ketones (0.24%), aldehyde (0.25%) and ethers (0.07%) (Figure 2). By comparing the results to *T. zygis* EOs from other regions of Morocco and around the world, chemical composition differences can be seen. Indeed, in Morocco, the essential oil of the same species of Krouchen, (Middle Atlas of Morocco) was dominated mainly by thymol (33.02%), *o-*cymene (32.02%) and (*E*)-β-ocimene (11.90%) [26]. In the Aknoul region (Taza region), the chemical composition was marked by the presence of thymol (37.5%), γ-terpinene (29.7%), and *p*-cymene (12.1%) [27]. In Europe, *T. zygis* from Northern Portugal was mainly composed of thymol (23.8%), geraniol (18.2%), geranyl acetate (16.3%) and *p*-cymene (13.6%) [28]. The essential oils of several samples of *T. zygis* from Spain studied by Richard et al. (1985), consist mainly of thymol (1.1 to 30.7%) or carvacrol (6.5 to 42.9%) accompanied by other constituents such as *p*-cymene (23.3 to 28.5%), caryophyllene oxide (1.5 to 9.8%), linalool (1.5 to 4%) and thymol methyl ether (0 to 4.5%) [8]. Older studies on the subspecies *gracilis* revealed the presence of phenolic chemotypes, mainly thymol or carvacrol [29,30,31,32,33,34]. We can conclude that the EO of *T. zygis* was characterized by a very interesting chemical composition. Concerning the EO of *T. willdenowii*, the chemical composition was marked by, oxygenated monoterpenes (32.81%), hydrocrboned sesquiterpene (31.61%), oxygenated sesquiterpene (13.35%), and hydrocarbon monoterpenes (8.95%), with the dominance of carvacrol (16.19%), geranyl acetate (8.35%), caryophyllene oxide (6.90%), camphor (5.99%), and (*E*)-caryophyllene (5.59%), in addition to other compounds at relatively low percentages such as borneol (4.74%) and β-elemene (3.96%). Comparing our findings with those of other researchers, we found that the EO collected from the Col du Zad in the region of Khenifra was rich in compounds other than monoterpenes such as terpenyl acetate (26.99%). *T. willdenowii* EO collected in Annzala of the region of Midelt at an altitude of (1605 m) was rich in oxygenated monoterpenes represented by camphor (24.99%) [18].

The EO of *T. zygis* seems to be characterized by a very interesting chemical composition, with chemical compounds in common (α-pinene, limonene, camphor, and carvacrol) and other different ones like germacrene D, which is present only in *T. willdenowii*. The latter composition is composed of 33.64% of alcohol, 40.58% of hydrocarbons, 7.82% of epoxides, 6.61% of ketones, 9.02% of esters and 2.32% of acids (Figure 2). This great variety and variability in the chemical composition of thyme essential oils is related to Moroccan geological and ecological diversity [35,36] which can even specify them from those from other regions all over the planet [37,38]. The presence or absence of a chemical component at any stage of development is solely controlled by the plant’s genetic history, but its concentration is influenced by both genetics and environmental variables [39].

Essential oils are likely to include thousands of molecules. A single essential oil may contain dozens, if not hundreds of distinct chemical components in wildly varied quantities. Some essential oils, on the other hand, may possess a nearly pure molecule such as Wintergreen (*Gaultheria procumbens* L.) which contains up to 99 percent methyl salicylate [40]. Many studies have made it possible to study the factors causing the chemical composition of thyme essential oil to vary, such as (i) harvest period (Several studies showed that the concentration of phenols (thymol and carvacrol) varies inversely with that of their precursors (*p*-cymene and *γ*-terpinene). Phenols are at their maximum level during the flowering period (June in the Northern hemisphere) and they are at their minimum during November / December period [41,42]. A study of the 1,8-cineole chemotypes in Spain found a peak level of 1,8-cineole during the growth phase of the plant [43]. The essential oil yield reaches its maximum during the period of full flowering.) (ii) Quality of the soil (which can affect the yield and quality of the essential oil) as the yield is better on calcareous soil than on sandy soil. Thymol production will be greater when the plant grows in sandy soil and less in clay soil. Limestone gives an intermediate proportion of thymol [44].)

In summary, many factors can influence the chemical composition of thyme essential oil. Regardless of the chemotype considered, the yield will be maximum at the time of flowering since physiological factors such as the stage of development of the plant and the nature of the secretory structures determine the quantity and quality of the essential oil produced [45]. Essential oils are generally more abundant in young organs. Many works have also shown the existence of a correlation between the qualitative composition of the essential oil and geographical variation [46,47]. Plant stress phenomena such as drought frequently alter the hormonal balance of the plant and modify the activity of many enzymes, as well as the expression of the genome [48].

### 2.4. Antioxidant Activity of the Essential Oils of the Two Thymes 

**Figure 3:**
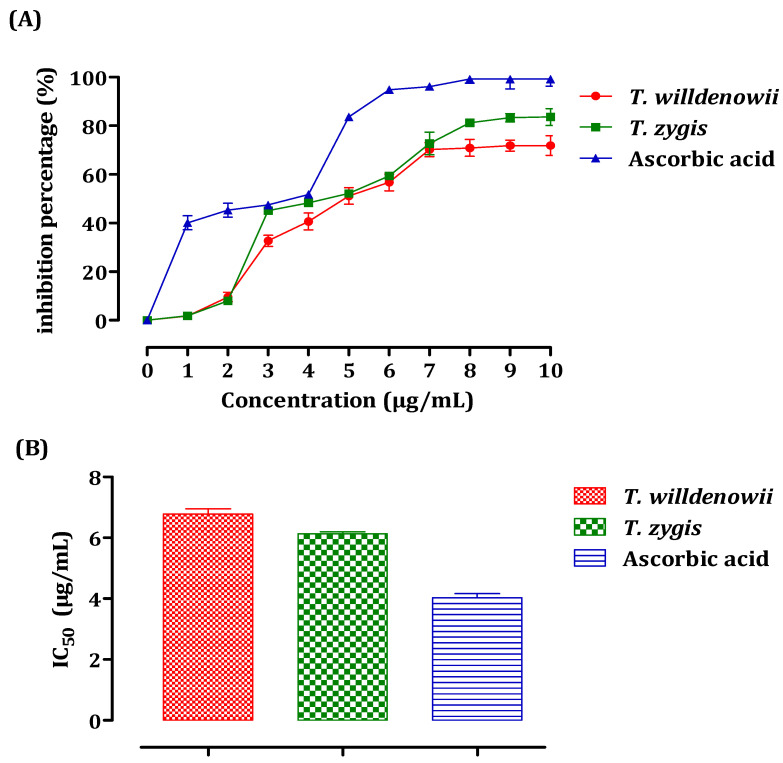
DPPH Free radical scavenging activity of *T. zygis*, *T. willdenowii*, and ascorbic acid. Inhibitory percentage (**A**) and IC_50_ (**B**) against the DPPH Free radical. data are presented as mean ± SD, the experiment was performed in a minimum of 2 replicates *T. zygis*: *Thymus zygis*, *T. willdenowii*: *Thymus willdenowii*.

The antiradical activity of essential oils was measured using spectrophotometry at 517 nm with the DPPH radical following the reduction of this radical accompanied by a change of color from violet to yellow. The results obtained made it possible to plot the curve of the inhibition percentage (%) as a function of the concentrations of essential oils (Figure 3A). These results show that the percentage of the free radical inhibition increased with the increase in the concentration of the EO, whether it is vitamin C (used as a positive control) or the essential oils of both thyme species. For all the concentrations tested, the inhibition percentage of vitamin C was greater than that of the two essential oils according to the IC_50_ values (Figure 3B). The value of IC_50_ is inversely related to the antioxidant capacity of a compound. It expresses the number of antioxidants necessary to decrease the concentration of the free radical by 50%. This concentration is determined graphically and expressed in μg/mL. *T.* *zygis* showed the greatest capacity for trapping DPPH, with an IC_50_ of the order of 6.13 ± 0.11 μg/mL against 6.78 ± 0.30 µg/mL noted for *T. willdenowii*. The antioxidant activity of the essential oils of *T. zygis* and *T. willdenowii* were greater than that obtained by Amarti et al. in their study concerning four essential oils of Moroccan thyme (*T. capitatus*, *T. ciliatus*, *T. bleicherianus*, and *T. algeriensis*), with an IC_50_ equal to 69.04 μL/mL, 74.025 μL/ mL, 77.24 μL/mL, 745 μL/mL, respectively [49].

**Figure 4:**
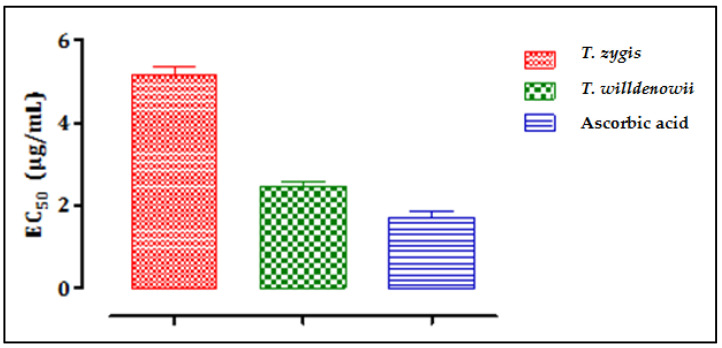
The effective concentrations (EC_50_) of *T. zygis*, *T. willdenowii* and ascorbic acid; data are mean ± SD, experiment was performed in minimum 2 replicates *T. zygis*: *Thymus zygis*, *T. willdenowii*: *Thymus willdenowii*.

Figure 4 shows the results of the evaluation of the antioxidant activity of essential oils of *T. zygis* and *T. willdenowii* by the iron reduction method (FRAP), characterized by the reduction of ferric iron Fe^3+^ (yellow) to ferrous iron Fe^2+^ (blue-green). *T. zygis* showed greater iron-reducing activity than that of *T. willdenowii* but less than that of ascorbic acid, which gave a greater iron reduction. The antioxidant activity of the two essential oils is expressed by determining the effective concentration (EC_50_) (Figure 4) which corresponds to an absorbance equal to 0.5.

From the results, we can deduce that the inhibition concentrations (IC_50_) to reduce 50% of the Fe^3+^ ions were 5.17 µg/mL, 2.46 µg/mL, and 1.76 µg/mL, for *T. willdenowii*, *T. zygis*, and ascorbic acid, respectively. There is a correlation between the chemical composition and the biological activity observed, especially with the high levels of carvacrol known for its high antioxidant potential. Indeed, several studies have demonstrated the superiority of the antioxidant power of essential oils with phenolic chemotypes (Carvacrol, thymol) [50,51]. Phenolics operate as reducing agents, hydrogen donors, and single oxygen donors due to their redox properties [52]. Nonetheless, other minor compounds can interact directly or in a synergistic or antagonistic manner to create a mixture endowed with more powerful activity. However, the antioxidant activity of the majority of compounds tested separately gives inferior results compared to the activity of the whole of the essential oil [53]. The two essential oils of the two thymes examined to reduce oxidation caused by free radicals demonstrated a potential to be effective against cancer and anti-infectious diseases and suggest that *Thymus* is a strong antioxidant that can be used as a natural antioxidant.

### 2.5. Antibacterial Activity of T. zygis and T. willdenowii EOs

#### 2.5.1. Antibiotic Sensitivity Test

The antibiotic sensitivity profiles of the strains indicated in Table 4 and Table 5 were carried out according to the recommendations of the French Society of Microbiology and The European Committee on Antimicrobial Susceptibility Testing (EUCAST) [54]. From the antibiogram, we can conclude that *Enterobacter cloacae* exhibited resistance to Ticarcillin, Ofloxacin, Amoxicillin + Clavulanic acid, Colistin, and Amoxicillin. As for the *Staphylococcu aureus*, *Salmonella Typhi*, and *Shigella dysenteriae* strains, they were sensitive to all the antibiotics tested except *E. coli* which showed resistance to Ticarcillin and Cefalexin (Table 5). At the same time, *Acinetobacter baumannii* demonstrated complete resistance to all tested antibiotics. The strain *A. baumanii* is the most frequently encountered species in human infections. A study conducted on 754 strains, mainly from intensive care units (50.53%), showed very high resistance to beta-lactams (91%), Cefotaxime (50.3%), Ceftazidime and Imipenem (42.6%). Resistance to aminoglycosides ranged from 17.9% for netilmicin to 72.1% for gentamicin. Resistance to Ciprofloxacin was 65.8% and to trimethoprimesulfamethoxazole 75.8% [55]. This may be due to the higher resistance of Gram-negative bacteria due to the complexity of their cell wall, containing a double membrane in opposition to the single glycoprotein/teichoic acid membrane of Gram-positive bacteria ) [56]. The bacterial strains chosen for this research are of great interest in the clinical and health fields. Their increasing resistance to antibiotics has prompted further research into new and more effective natural products. Some strains of *E. coli* are virulent and can specifically trigger spontaneous infections of the gastrointestinal tract or urinary tract and even neonatal meningitis in humans or certain animal species. Other strains belonging to the symbiotic flora can also cause a variety of opportunistic infections, particularly in individuals with weakened immune defenses [56]. *S. aureus* is the cause of meningitis, osteomyelitis and diarrhea [57]. *E. cloacae* is a major pathogen within the genus Enterobacter. It is an opportunistic pathogenic Gram-negative bacillus mostly involved in nosocomial infections in compromised patients [58]. The bacterial pathogen *S.* *typhi* causes a serious systemic disease called typhoid, which is a major public health problem of global importance [59]. Shigellosis is an acute invasive intestinal infection caused by bacteria belonging to the genus *Shigell*a; it is clinically manifested by diarrhea often bloody [60]. Antibiotic susceptibility testing was performed to demonstrate the power of the essential oils against resistant strains. The diameter of the inhibition zones was considered resistant for diameters less than 8 mm, and sensitive for those over 20 mm.

#### 2.5.2. Antibacterial Activity of *T. zygis* and *T. willdenowii* Essential Oils

The antibacterial activity of *T. zygis* and *T. willdenowii* EOs was evaluated by the disk diffusion method and by tests to determine the MIC and MBC. For the disk diffusion method, EOs are considered to be active when they induce an inhibition zone greater than or equal to 12 mm [61,62]. The average inhibition diameters, generated by the EOs tested on the different bacterial strains tested were presented in Table 6 given below. Statistical analysis of the results showed that the diameters of inhibition were significantly different for EOs (*p* < 0.05). According to the antibacterial results tests, all the bacteria demonstrated significant inhibition zones when tested against the two EOs. *T. zygis* EO exhibited the most powerful activity against all the studied bacteria, while that of *T. willdenowii* recorded moderate activities against *S. dysenteriae* and *S.* Typhi. With an inhibition diameter between 75 mm and 84 mm for a concentration of 2 µL/mL up to 12 µL/mL, *S. aureus* was shown to be the most sensitive to *T. zygis* EO. In addition, Gram-positive bacteria are more sensitive to *T. zygis* essential oil action compared to Gram-negative bacteria. It is known that the structure of the cell wall of Gram-positive bacteria makes them vulnerable to the action of essential oils [4]. These different results between the two essential oils could be explained by the bioactivity of the chemical compounds of each oil, the functional groups of the major compound (alcohols, phenols, aldehydes), and the synergistic effects between the components. Thus, the most effective chemical compounds which have a broad spectrum of antimicrobial action are phenols (Thymol, carvacrol, and eugenol), alcohols (*α*-terpineol, terpinene-4-ol, menthol, geraniol, linalool), aldehydes (geraniol, citral, and neral), and ketones (carvone, pulegone, and camphor) [63,64]. Analysis of the results related to the MIC and MBC of the two EOs revealed their great bactericidal power (Table 7). Nonetheless, Gram-positive bacteria were found to be susceptible to EOs as well as Gram-negative with a difference between MIC and MBC. Indeed, *E. coli* was shown to be sensitive from the concentration of 2 μL/mL towards the EO of *T. zygis* whereas *S. dysenteriae* was resistant up to the concentration of 10 μL/mL of *T. willdenowii* EO. The chemotype carvacrol found in *T. zygis* essential oil with a concentration of 52.2% of the total essential oil remains the most effective compared to the other *T. willdenowii* essential oil which contains only 16.2% of carvacrol. The high carvacrol content of *T. zygis* essential oil could explain the bactericidal effect on the different strains. Phenols are, due to the acidic nature of their hydroxyl substituent, considered as the most active compounds on bacteria [65] knowing that the total chemical composition of the EO of this thyme is dominated by 69.53% of alcohols. Indeed, alcohols are particularly active against bacterial strains, because they are soluble in aqueous media and cause significant damage to the cell walls of microorganisms [66]. Alcohols have bactericidal rather than bacteriostatic activity [67].

### 2.6. Antifungal Activity of T. zygis and T. willdenowii EOs

#### 2.6.1. Sensitivity of Fungal Strains

The antifungal susceptibility profiles of the strains indicated in Table 8 were carried out according to the recommendations of EUCAST. Both strains *C. glabrata* and *C. albicans* were sensitive to the antifungal agent (Fluconazole) while *Candida* spp., *A. fischeri*, and *F. solani* were resistant. The candidas particularly responsible for the infection, which typically occurs in patients with impaired immune function or who have had a mucosal invasive procedure (ANOFEL, 2014). The prevalence of yeasts of the genus *Candida* resistant to first-generation triazole antibiotics is low. Primary resistance has been described in less than 2.5% of cases for fluconazole and in less than 9% of cases for litraconazole (5). *C. albicans* is most often susceptible, *C. glabrata* is often susceptible-dose dependent [68]. Whereas fluconazole is not active in vitro against filamentous fungi such as *Aspergillus* spp. (MIC of 64 mg/L) [69]. Fungi of the genus *Fusarium* are resistant to the majority of available human antifungal agents: they are resistant in vitro to flucytosine, to first-generation triazoles (fluconazole, itraconazole) [70].

#### 2.6.2. Antifungal Activity of Essential Oils

**Figure 5:**
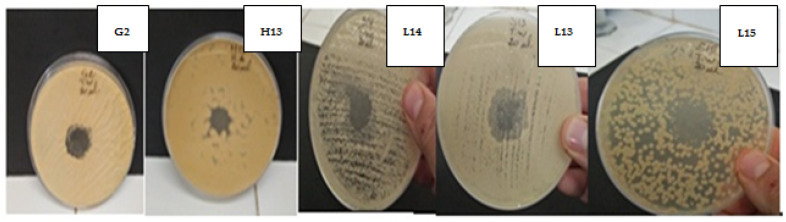
Antifungal activity of the essential oil of *T. willdenowii* tested on *C. albicans* (L13), *C. glabrata* (L14), *Candida* spp. (L15), *F. solani* (H13), and *A. fischeri* (G2).

The genus *Thymus* was used against bacteria and fungi in the traditional pharmacopeia [71]. The volatile oils studied showed very good antifungal power, referring to the reading established by Meena and Sethi, and Ponce et al. [72,73]. The diameter of the inhibition zone is noted as resistant for diameters less than 8 mm, and sensitive for diameters greater than 20 mm. From Table 9 it can be seen that the EO of *T. zygis* showed the best inhibition diameters compared to the control (fluconazole) while *T. willdenowii* demonstrated a similar (*F. solani*) and even better inhibition zone (*A. fischeri*) when also compared to fluconazole the molds and yeasts showed a sensitivity to the concentration of 20 µL of the two Eos and the inhibition zones were of the order of 40 mm for *C. albicans*, 29 mm for *C.* *glabrata*, 27 mm for *Candida* spp., 18 mm for *F. solani* and 40 mm for *A. fischeri*, when using *T. zygis* EO, all diameters were greater than the control values used (fluconazole). The EO of *T. willdenowii*, when used, recorded smaller diameters than the other EO (*T. zygis*) (Table 9 and Figure 5). Regarding the MIC and MFC (Table 10), the Eos of the two thymes showed significant fungicidal activity on both yeasts and molds, even against strains resistant to the control, *Candida* spp., *A. fischeri* and *F. solani*. The inhibitory effect of *T. zygis* EO was manifested at 20 µL/mL while that of *T. willdenowii* was active at 30 µL/mL. MFC/MIC values are equal to 1.

This great activity can be linked to the presence of predominant phenolic compounds such as carvacrol which is known for its antimicrobial properties [74,75]. The mechanism of action of Eos remains controversial; some studies suggest that these components can enter the microorganism and react with active sites of enzymes and or interfere with cell metabolism, but several proposals lean towards disruption of cell membranes and pro-oxidant cytotoxic effects [76]. Essential oils’ action is frequently affected by their hydrophobic characteristic, which enables them to permeate the bacterial cell membrane’s phospholipidic double layer. This may cause a change in membrane conformation, a chemiosmotic disturbance, and ion leakage (K+) [77].

Some essential oil phenolic compounds interact with membrane proteins of microorganisms, such as the ATPase enzyme, either directly on the hydrophobic portion of the protein or by interfering with protons translocation through the membrane, inhibiting the phosphorylation of ADP. Decarboxylation of amino acids in *E. aerogenes* has also been shown to be inhibited [78]. The results showed that the inhibition diameters are significantly different for EOs (*p* < 0.05).

Because of its anti-infectious properties, the EO of the two examined thymes may be likened to antibiotics and antifungals. This antimicrobial action on yeasts, as well as Gram (+) and Gram (−) bacteria that are multi-resistant to antibiotics, may help in the battle against infectious illnesses and could lead to the use of these EOs in the pharmaceutical and agrifood industries.

## 3. Materials and Methods

### 3.1. Plant Material

**Figure 6:**
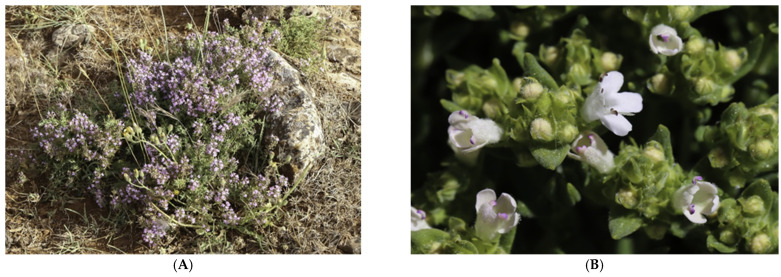
(**A**) *Thymus zygis* L. and (**B**) *Thymus willdenowii* Boiss.

Samples of the aerial part (stems, leaves, and flowers) of *T. zygis* (RTCBME11) and *T. willdenowii* (RTCBME12) (two wild thymes) were collected from two sites of the province of Ifrane (Middle Atlas of Morocco) whose geographical coordinates are: 31°42′07″ Nord, 6°20′57″ Ouest. This region is characterized by a Mediterranean mountain climate, with a wet and cold winter and a dry summer. Temperatures vary around 0 and 37 °C, and precipitation can reach 100 days per year, including 15 to 30 days of snow; the snow cover, 30–60 cm thick, can persist for more than 50 days in normal years. Wild *T. zygis* (Figure 6A) was collected in May (2019) in Azrou and wild *T. willdenowii* (Figure 6B) in May (2019). The identification of the two plant species was carried out at the Botanical and Plant Ecology Laboratory of the Scientific Institute of Rabat (Morocco). The aerial parts of the two plants were dried at room temperature for 15 days, protected from light, then cleaned and stored away from light and humidity.

### 3.2. Extraction of Essential Oils

Essential oils were extracted by hydrodistillation using a Clevenger-type apparatus for three hours [79]. The essential oil yield was determined in mL relative to 100 g of dry matter. The essential oil obtained was stored at 4 °C in the dark.

### 3.3. Physicochemical Analysis of the Essential Oils of the Two Thymes: Refractive Index, Brix Degree, and Density

Refractive index: This index was generally measured at 20 °C using a refractometer. A drop of essential oil was placed on the flat section of the glass prism then the value was given directly by the device.

Brix degree: The Brix degree principle was based on measuring the concentration (%) of all solids dissolved in the oil (sugar, salts, proteins, fatty acids, etc.). This parameter was measured by a refractometer device.

Density: The density was measured using a hydrometer consisting of a cylindrical, hollow, graduated glass tube ballasted with a lead shot.

### 3.4. Gas Chromatography Coupled with Mass Spectrometry Analysis of Essential Oils

The chromatographic analysis of the essential oils from the aerial part of the two plants was carried out on a gas chromatograph of the Thermo Electron type (Trace GC Ultra) coupled to a mass spectrometer of the Thermo Electron Trace MS system type (Thermo Electron: Trace GC Ultra; Polaris Q MS), fragmentation was carried out by the electronic impact of intensity 70 eV. The chromatograph was equipped with a DB-5 (5% phenyl-methyl-siloxane) type column (30m × 0.25mm × 0.25μm film thickness), a flame ionization detector (FID) powered by a mixture of He gas/Air. The temperature of the column was programmed at a rate of a rise of 4 °C/min from 50 to 200 °C for 5 min. The injection mode was split (leakage ratio: 1/70, flow rate mL/min), the carrier gas used was nitrogen with a flow rate of 1 mL/min. The identification of the chemical composition of the essential oils of the two plants was carried out based on the comparison of their Kováts index (KI) and Adams with those of the reference products known in the literature [80,81]. It was supplemented by a comparison of indices and mass spectra with different references [82,83]. The Kováts index compares the retention time of any product with that of a linear alkane of the same carbon number.

### 3.5. Antioxidant Activity of the Essential Oils

#### 3.5.1. DPPH Free Radical Scavenging Assay

The anti-free radical activity of the different essential oils of the two plants was established by the method based on DPPH* (1,1-diphenyl-2-picrylhydrazyl) as a relatively stable radical [84]. The DPPH* solution was prepared by dissolving 2.4 mg of DPPH* in 100 mL of ethanol. The EOs were prepared by dissolving them in ethanol (2 mg/mL). This stock solution will undergo a series of dilutions to have the following concentrations: (1; 2; 3; 4; 5; 6; 7; 8; 9; 10 µg/mL). The test was carried out by mixing 2 μL of the compound to be tested and 2.8 mL of DPPH* solution. These same concentrations were prepared with ascorbic acid (vitamin C) to serve as positive controls. All assays were performed in triplicates. The samples were then left in the dark for 30 min, the absorbances were measured at 517 nm.

(1) $$\mathrm{AA}\%=\frac{A\;\mathrm{control}-A\;\mathrm{sample}}{A\;\mathrm{control}\;}\times 100$$

AA%: Percentage of antioxidant activity.

A control: absorbance of the DPPH˙ radical solution

A sample: absorbance of the solution of the samples to be tested in the presence of DPPH.

#### 3.5.2. Ferricyanide FRAP Assay

The power of EOs to reduce ferric iron (Fe^3+^) present in the potassium ferricyanide complex to ferrous iron (Fe^2+^) was determined according to the method described by Zovko Koncić et al. [85]. In test tubes, 0.5 mL of EOs at different concentrations (a concentration range from 1 to 10 mg/mL) was mixed with 2.5 mL of a 0.2 M of phosphate buffer solution (pH = 6.6) and 2.5 mL of a solution of potassium ferricyanide K_3_Fe (CN)_6_ to 1%. The whole was incubated in a water bath at 50 °C for 20 min. Then 2.5 mL of 10% trichloroacetic acid was added to stop the reaction. The whole was centrifuged at 3000 revolutions for 10 min. Then 2.5 mL of the supernatant from each concentration was mixed with 2.5 mL of distilled water and 0.5 mL of the 0.1% aqueous FeCl_3_ solution. All assays were performed in triplicates. The absorbance of the reaction medium was read at 700 nm against a similarly prepared blank, with distilled water which makes it possible to calibrate the device (UV-VIS spectrophotometer). The positive control was represented by standard solutions (Ascorbic acid) whose absorbance was measured under the same conditions as the samples. The graph of the change in absorbance as a function of extract concentration was used to determine the median effective concentration (EC_50_) by linear regression.

### 3.6. Antibacterial and Antifungal Activity

#### 3.6.1. Selected Microbial Strains and Sensitivity Test

All microbial strains studied were clinical isolates: six bacterial strains (*Escherichia coli*, *Staphylococcus aureus*, *Acinetobacter baumannii*, *Shigella dysenteriae*, *Salmonella* Typhi, and *Enterobacter cloacae*) were chosen for their pathogenicity and their incrimination in human infections. The six bacteria belong to both, the Provincial Laboratory of Epidemiology and Environmental Hygiene in Ifrane, and the medical laboratory of the 20th August Provincial Hospital in the province of Ifrane. For the antifungal activity, five fungal strains including three yeasts (*Candida albicans*, *Candida glabrata*, and *Candida* spp.) and two molds (*Aspergillus fischeri*, and *Fusarium solani*) were tested. The five strains belong to the Laboratory of Ecology and Biodiversity of Wetlands, Moulay Ismail University, Faculty of Sciences in Meknes. The antibiotic and antifungal sensitivity profiles of the strains tested were carried out according to the recommendations of the French Society of Microbiology and EUCAST [86].

#### 3.6.2. Disc Diffusion Method

We used the solid-state diffusion method (Muller–Hinton medium) for antibacterial activity and (Sabouraud chloramphenicol medium) for antifungal activity. The antibacterial and antifungal assays we used are derived from those used by [87]. Initially, we filtered our oils through a Millipore filter with a pore diameter of 0.45 µm.

Bacterial and fungal suspensions were prepared from pure cultures in the exponential phase of growth by a standardized inoculum (10^5^ CFU mL) on MHA and Sabouraud chloramphenicol medium in sterile physiological water (EρS). Then from the prepared inoculum, we take 1 mL of each bacterial and fungal suspension, we spread it by flooding on the surface of a Petri dish containing MHA agar for bacteria and Sabouraud chloramphenicol for fungi, the excess liquid is aspirated. We keep the Petri dish in the septic zone of the Bunsen burner until it becomes dry, then we place the sterile blotting paper discs in the center, and we place on it the volumes of 2 µL, 4 µL, 6 µL, 8 µL, 10 µL, 12 µL, of the essential oil (EO) to be tested. For the bacteria, a sterile disc flooded with 2 µL of DMSO was used as a negative control (as DMSO was used as an emulsifying agent in the MIC test). The plates were then incubated at 37 °C for 18–24 h. After incubation, the reading is taken by measuring the diameter of the inhibition zone.

For the antifungal assays, each of the 6 mm diameter sterile Whatman paper discs is impregnated with 20 µL of each EO on the surface of the petri dish medium. A negative control disc flooded with 20 µL of sterile physiological water was used for the fungi.

#### 3.6.3. Minimum Inhibitory Concentration (MIC)

This technique derives from those used by [88], it consists of inoculating a standardized inoculum (10^5^ CFU/mL) with a range of increasing concentrations of essential oil. After incubation, observation of the range allows the determination of the Minimum Inhibitory Concentration (MIC), which corresponds to the lowest concentration of essential oil for which bacterial growth is no longer visible, in vitro (no growth but 100% of the bacteria surviving) 2 µL of DMSO is transferred to the 10 hemolysis tubes containing the culture medium (1 mL) for each bacterial strain. Each concentration of essential oil (EO) is introduced into a hemolysis tube. The 10 chosen concentrations of EO are 2 µL/mL, 4 µL/mL, 6 µL/mL, 10 µL/mL, 12 µL/mL, 14 µL/mL, 16 µL/mL, 18 µL/mL, 20 µL/mL, and 22 µL/mL. Two repetitions were made for each concentration. Then a volume of 6 µL of the bacterial suspension with a concentration of 10^5^ CFU/mL was taken and placed in each of the previous tubes and then 2 µL of DMSO was added for each bacterial strain.

For the fungi the concentrations chosen for the EO are 10 µL/mL, 20 µL/mL, 30 µL/mL, 40 µL/mL, 50 µL/mL and 60 µL/mL.

We used dimethyl-sulfoxide amide (DMSO) as an emulsifying agent (E) because of its effectiveness as a solubilizing agent for essential oils with a low concentration (2 µL) that does not influence the antibacterial quality of the tested essential oil.

For each oil, two controls were made; one containing the culture medium (1 mL) plus a bacterial strain and the other containing the culture medium (1 mL) plus 2 µL of the essential oil alone. The MIC is deduced from the first tube where bacterial growth is inhibited.

#### 3.6.4. Minimum Bactericidal Concentration (MBC) and Minimum Fungicidal Concentration (MFC)

**Figure 7:**
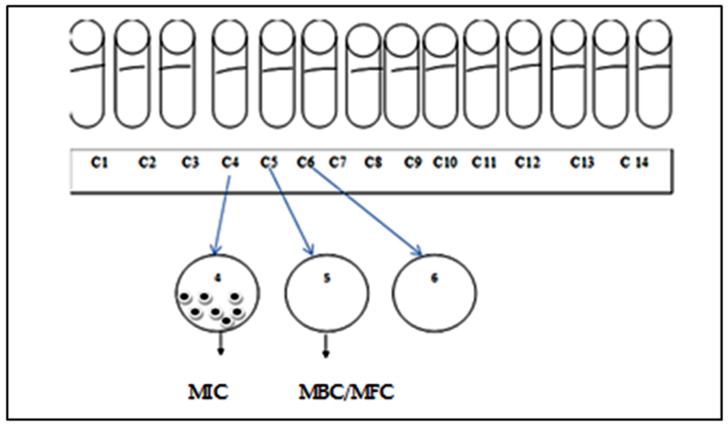
Determination of the Minimum Bactericidal Concentration (MBC) and Minimum Fungicidal Concentration (MFC) of essential oils. In this example the MIC is C4 where the strains are alive but inhibited from growth in the tube and the MBC or MFC is C5 where the strains are totally killed by the EOs.

The Minimum Bactericidal Concentration (MBC) and the Minimum Fungicidal Concentration (MFC) are the lowest concentrations of essential oil capable of killing more than 99.9% of the initial inoculum (i.e., less than 0.01% of survivors). It defines the bactericidal effect of an essential oil. The volume range used for the MIC determination was used to determine the BMC of the essential oil to be tested. Samples were taken from the tubes used to determine the MIC of each strain using a loop and then inoculated onto Petri dishes containing the culture medium. The inoculated plates were incubated for 24 h at a temperature of 37 °C. The MBC and MFC of the essential oil are deduced from the first plate free of bacteria or fungi (Figure 7).

### 3.7. Statistical Analysis

The evaluation of the effect of the essential oils (diameter of inhibition) tested on the growth of fungi and bacteria as well as the antioxidant activity was carried out by an analysis of variance (ANOVA). The Means and standard deviations were calculated using the Graph Pad Prism 5 for windows, analysis of variance (ANOVA) was used to calculate the significance with Tukey as a post hoc test. A 5% probability threshold was used for the comparison of the means.

## 4. Conclusions

This work aimed to study the antimicrobial and antioxidant activities of the essential oils of *T. zygis* and *T. willdenowii*. From the results obtained it appears that these two plants have virtues that can justify their use in traditional medicine. The composition of the EOs explains partially the powerful observed activities (antioxidant and antimicrobial), especially the role of the major compounds in the overall bioactivity. The obtained antimicrobial results require more attention as they showed excellent activities even against resistant strains. From all of the above, we confirm the traditional use of those plants’ EO against infectious disease and we encourage further, more advanced tests for more personalized applications either in the clinical or the industrial sectors.

## Figures and Tables

**Table 1:** The Eos yields of the two selected thyme species.

Harvest Site	EO	Yield (%)
Azrou	*T. zygis*	5.25 ± 0.01
Ifrane	*T. willdenowii*	3.00 ± 0.02

**Table 2:** Refractive index, degree Brix, and essential oils density of *T. zygis* and *T.* *willdenowii*.

	*T. zygis*	*T. willdenowii*
Density	0.92 ± 0.05	0.91 ± 0.05
Refractive index	1.50 ± 0.05	1.33 ± 0.04
Degree degree (%)	85.44 ± 0.05	76.62 ± 0.05

**Table 3:** Chemical composition of the thymes EOs.

No.	Compounds	Kováts Index (KI)	Molecular Formula	Area%	
				*T. willdenowii*	*T. zygis*
1	*α*-Pinene	939	C_10_H_16_	0.59	0.62
2	Camphene	954	C_10_H_16_	0.96	0.78
3	β-Pinene	979	C_10_H_16_	-	0.06
4	1-Octen-3-ol	979	C_8_H_16_O	-	0.20
5	Myrcene	990	C_10_H_16_	2.43	0.27
6	3-δ-Carene	1002	C_10_H_16_	-	0.29
7	*p*-Cymene	1024	C_10_H_14_	1.78	-
8	*o*-Cymene	1026	C_10_H_14_	-	23.14
9	Limonene	1029	C_10_H_16_	2.16	0.28
10	1,8-Cineole	1031	C_10_H_18_O	1.77	0.18
11	*(Z)-*β-ocimene	1037	C_10_H_16_	0.42	-
12	*γ*-Terpinene	1059	C_10_H_16_	0.61	1.98
13	*Cis*-Linalool oxide	1072	C_10_H_18_O_2_	-	0.13
14	*Trans*-Linalool oxide	1086	C_10_H_18_O_2_	-	0.25
15	Linalool	1096	C_10_H_18_O_2_	1.78	2.40
16	Camphor	1146	C_10_H_16_O2	5.99	0.16
17	Borneol	1169	C_10_H_18_O	4.74	3.30
18	Terpinen-4-ol	1177	C_10_H_18_O	1.06	0.35
19	α-Terpineol	1188	C_10_H_18_O	-	0.10
20	Pulegone	1237	C_10_H_16_O	-	0.08
21	Carvacrol methyl ether	1244	C_10_H_16_O	-	0.07
22	Thymol	1290	C_10_H_14_O	1.28	9.68
23	Carvacrol	1299	C_10_H_14_O	16.19	52.2
24	Geranyl acetate	1381	C_12_H_20_O_2_	8.35	-
25	β-Bourbonene	1388	C_15_H_24_	2.48	-
26	β-Elemene	1390	C_15_H_24_	3.96	-
27	(*E*)-Caryophyllene	1419	C_15_H_24_	5.59	0.99
28	β-YLangene	1420	C_15_H_24_	0.51	-
29	γ-Elemene	1436	C_15_H_24_	0.82	-
30	Germacrene D	1481	C_15_H_24_	16.51	-
31	α-Murolene	1500	C_15_H_24_	-	0.09
32	γ-Amorphene	1512	C_15_H_24_	0.87	0.07
33	γ-Cadinene	1513	C_15_H_24_	0.87	-
34	Spathulenol	1578	C_15_H_24_O	0.96	0.18
35	Caryophyllene oxide	1583	C_15_H_24_O	6.90	1.06
36	*Allo*-Aromadendrene epoxide	1640	C_15_H_24_O	0.92	-
37	Caryophylla-4(12),8(13)-dien-5β-ol	1640	C_15_H_24_O	-	0.09
38	*Epi*-α-Cadinol	1640	C_15_H_26_O	-	0.09
39	Cubenol	1646	C_15_H_26_O	0.55	-
40	Eudesmol	1650	C_15_H_26_O	0.61	-
41	Cedr-8(15)-en-10-ol	1652	C_15_H_24_O	-	0.20
42	α-Cadinol	1654	C_15_H_18_	1.32	-
43	Cadalene	1676	C_15_H_18_	-	0.23
44	Germacra-4(15),5,10(14)-trien-1α-ol	1686	C_15_H_24_O	2.09	-
45	hexahydrofarnesyl acetone	1932	C_18_H_36_O	0.62	-
46	Cembrene C	1940	C_20_H_18_	0.69	-
47	Hexadecanoic acid	1960	C_16_H_32_O_2_	2.31	-
48	*Cis*-Totarol, methyl ether	2208	C_21_H_32_O	-	0.07
Oxygenated monoterpenes				32.81	68.7
Hydrocarbon monoterpenes				8.95	27.55
Hydrocarboned sesquiterpene				31.61	1.4
Oxygenated sesquiterpene				13.35	1.85
Lignar esters				8.35	-
Others				3.62	0.27
Total				98.69	99.84

**Table 4:** Antibiotic sensitivity test for *A. baumannii* and *E. cloacae*.

ATB	*A. baumannii*	ATB	*E. cloacae*
TIC75	6 ± 00 (R)	TIC75	6 ± 00 (R)
CAZ30	6 ± 00 (R)	CAZ30	20 ± 00 (S)
MEM10	6 ± 00 (R)	OFX5	6 ± 00 (R)
TIM85	6 ± 00 (R)	AMC3	6 ± 00 (R)
IPM10	6 ± 00 (R)	IPM10	27 ± 0.1 (S)
CT50	6 ± 00 (R)	CT50	6 ± 00 (R)
TOB10	6 ± 00 (R)	FOX30	21 ± 00 (S)
CIP5	6 ± 00 (R)	AML10	6 ± 00 (R)
TE30	6 ± 00 (R)	CN15	22 ± 0.2 (S)
CN15	21.5 ± 0.1 (S)	AK30	19 ± 00 (I)
AK_30_	6 ± 00 (R)		
PRL_75_	6 ± 00 (R)		

S: sensitive at standard dose, R: resistant, ATB: antibiotics, TOB: Tobramycin, TIC: Ticarcillin, AML: Amoxicillin, FOX: Cefoxitin, CT: Colistin, CIP: Ciprofloxacin, AK: Amikacin, IPM: Imipenem, CAZ: Ceftazidime, PRL: Piperacillin, TE: Tetracycline, CN: Cefalexin, MEM: Meropenem, TIM: Ticarcillin + Clavulanic acid, OFX: Ofloxacin, Antibiotic disc load was in µg.

**Table 5:** Antibiotic sensitivity test for *S. aureus*, *E. coli*, *S.* Typhi, and *S. dysenteriae*.

ATB	*S. aureus*	ATB	*E. coli*	*S.* Typhi	*S. dysenteriae*
CIP5	23 ± 0.1 (S)	CT50	20 ± 0.1 (S)	20 ± 0.1 (S)	21 ± 0.2 (S)
VA30	26 ± 0.3 (S)	MEM10	21 ± 0.2 (S)	22 ± 0.2 (S)	23.5 ± 0.1 (S)
TE30	24.5 ± 0.2 (S)	TIC75	06 ± 00 (R)	21.5 ± 00 (S)	20.5 ± 0.3 (S)
CN15	21 ± 0.1 (S)	AK30	20 ± 0.1 (S)	22 ± 0.2 (S)	22 ± 0.1 (S)
MY15	30 ± 0.1 (S)	C30	27 ± 00 (S)	29 ± 00 (S)	20 ± 00 (S)
E15	20 ± 00 (S)	PRL75	21 ± 00 (S)	21 ± 00 (S)	21 ± 00 (S)
CAZ30	22 ± 0.3 (S)	IPM10	23 ± 0.2 (S)	21 ± 0.3 (S)	23 ± 0.2 (S)
TOB10	21.5 ± 0.1 (S)	CIP5	20 ± 0.1 (S)	20.5 ± 0.2 (S)	30.5 ± 0.1 (S)
SXT25	20 ± 0.2 (S)	AMC30	21 ± 0.2 (S)	21 ± 0.1 (S)	20 ± 0.2 (S)
FD10	23 ± 0.4 (S)	CN15	06 ± 00 (R)	20 ± 0.2 (S)	21 ± 0.1 (S)
FOX30	22 ± 0.1 (S)	CAZ30	20 ± 0.1 (S)	23.5 ± 0.1 (S)	20.5 ± 0.2 (S)
RD30	25 ± 00 (S)	CRO30	21 ± 00 (S)	22 ± 00 (S)	24 ± 00 (S)
OFX5	20 ± 0.1 (S)	CTX30	20 ± 0.2 (S)	21 ± 0.1 (S)	23 ± 0.2 (S)

S: sensitive at standard dose, R: resistant, ATB: antibiotics, CRO: Ceftriaxone, TOB: Tobramycin, AML: amoxicillin, FOX: Cefoxitin, C: Chloramphenicol, CT: Colistin, AMC: Amoxicillin + clavulanic acid, CIP: Ciprofloxacin, AK: Amikacin, IPM: Imipenem, CAZ: Ceftazidime, PRL: Piperacillin, SXT: Trimethoprim + sulfamethoxazole, TE: Tetracycline, CN: Cefalexin, MEM: Meropenem, TIM: Ticarcillin + clavulanic acid, OFX: Ofloxacin, VA: Vancomycin, MY: Lincomycin, FD: Fusidic acid, RD: Rifampicin, CTX: Cefotaxime. Antibiotic disc load was in µg.

**Table 6:** Antibacterial activity of *T. zygis* and *T. willdenowii* EOs.

Essential Oils Concentration Tested (µL/mL)	Essential Oils Inhibition Diameter (mm)											
	*S. aureus*		*E. coli*		*S.* Typhi		*A. baumannii*		*E.* *cloacae*		*S. dysenteriae*	
	*Tz*	*Tw*	*Tz*	*Tw*	*Tz*	*Tw*	*Tz*	*Tw*	*Tz*	*Tw*	*Tz*	*Tw*
2	75 ± 00 ***	33 ± 0.2 ***	54 ± 00 ***	15 ± 00 ^ns^	20 ± 00 *	06 ± 00 ^ns^	71.5 ± 0.1 ***	30 ± 00 **	60.1 ± 0.1 ***	14 ± 00 ^ns^	6 ± 00 ^ns^	6 ± 00 ^ns^
4	84 ± 0.2 ***	33.8 ± 0.1 ***	60 ± 00 ***	16.3 ± 0.1 ^ns^	24.5 ± 0.3 *	6 ± 00 ^ns^	72 ± 00 ***	35 ± 00 **	64.5 ± 0.4 ***	15.5 ± 1.2 ^ns^	18 ± 0.1 ^ns^	6 ± 00 ^ns^
6	84 ± 0.1 ***	38 ± 0.3 ***	71 ± 00 ***	18 ± 00 *	30.1 ± 0.2 **	13.5 ± 0.1 ^ns^	76.3 ± 0.2 ***	43.6 ± 0.1 ***	71.3 ± 0.3 ***	21 ± 00 *	37.5 ± 0.9 **	6 ± 00 ^ns^
8	84 ± 0.6 ***	42 ± 00 ***	82.2 ± 0.2 ***	21.4 ± 0.2 *	50 ± 00 ***	16 ± 00 ^ns^	78.1 ± 0.1 ***	51 ± 00 ***	77 ± 00 ***	23 ± 00 *	40 ± 00 ***	13 ± 00 ^ns^
10	84 ± 0.3 ***	47.2 ± 0.1 ***	84 ± 00 ***	22.1 ± 0.3 *	52 ± 00 ***	18.9 ± 1.3*	80.7 ± 1.4 ***	56 ± 00 ***	78.9 ± 0.1 ***	35 ± 00 *	48.6 ± 0.4 ***	15 ± 00 ^ns^
12	84 ± 00 ***	48 ± 00 ***	84 ± 00 ***	23 ± 00 *	57.5 ± 00 ***	23 ± 00 *	81 ± 00 ***	60 ± 00 ***	82 ± 00 ***	38 ± 00 *	51 ± 00 ***	18 ± 00 *
2(DMSO)	06 ± 00	06 ± 00	06 ± 00 ***	06 ± 00	06 ± 00	06 ± 00	06 ± 00	06 ± 00	06 ± 00	06 ± 00	06 ± 00	06 ± 00

The experiment was performed in minimum 2 replicates with *** *p* ≤ 0.001, ** *p* ≤ 0.01, * *p* ≤ 0.05; ^ns^: not significant compared to control DMSO. *Tz*: *T. zygis*; *Tw*: *T. willdenowii.*

**Table 7:** Determination of MIC and MBC of *T. zygis* and *T. willdenowii* EOs.

Bacteria	*T. zygis*		*T. willdenowii*	
	MIC µL/mL	MBC µL/mL	MIC µL/mL	MBC µL/mL
*E. coli*	02 ± 0.002 *	02 ± 0.001 *	04 ± 0.006 *	04 ± 00 ^ns^
*S. aureus*	02 ± 0.0009 *	02 ± 0.004 *	04 ± 00 ^ns^	04 ± 0.01 **
*S*. Typhi	04 ± 0.003 *	04 ± 00 ^ns^	06 ± 00 ^ns^	06 ± 00 ^ns^
*A. baumannii*	02 ± 0.001 *	02 ± 0.001 *	04 ± 0.001 *	04 ± 0.012 **
*E. cloacae*	02 ± 0.007 *	02 ± 00 ^ns^	06 ± 0.008*	06 ± 0.005 *
*S. dysenteriae*	06 ± 0.0001 *	06 ± 0.0003 *	10 ± 00 ^ns^	10 ± 00 ^ns^

The experiment was performed in minimum 2 replicates with ** *p* ≤ 0.01, * *p* ≤ 0.05; ^ns^: not significant.

**Table 8:** Susceptibility test of fungal strains to antifungal.

Fungal Species	Antifungal: Fluconazole V = 20 µL
*C. glabrata*	S
*C. albicans*	S
*Candida* spp.	R
*A. fischeri*	R
*F. solani*	R

**Table 9:** Effect of the EO on the growth of yeasts and molds.

Species	C = 20 µL/mL				
	Volatile Oil Inhibition Diameter (mm)				
	*C. glabrata*	*C. albicans*	*Candida* spp.	*F. solani*	*A. fischeri*
*T. zygis*	40 ± 0.3 *	29 ± 0.5 ^ns^	27 ± 1.2 ^ns^	18 ± 1.3 ^ns^	40 ± 2.1 **
*T. willdenowii*	17 ± 0.1 ^ns^	19 ± 1.1 ^ns^	23 ± 0.1 ^ns^	12 ± 00 ^ns^	22 ± 00 *
*Fluconazole*	24.7 ± 0.1	23.7 ± 0.2	24 ± 0.6	16.3 ± 0.3	13.7 ± 02

The experiments were performed in a minimum of 2 replicates with ** *p* ≤ 0.01, * *p* ≤ 0.05; ^ns^: not significant compared to fluconazole.

**Table 10:** MIC and MFC values of *T. zygis* and *T. willdenowii* essential oils.

Fungal Species	*T. zygis*			*T. willdenowii*		
	MIC µL/mL	MFC µL/mL	MFC/MIC	MFC µL/mL	MFC µL/mL	MFC/MIC
*C. glabrata*	20 ± 0.001 *	20 ± 0.0009 *	1	30 ± 00 ^ns^	30 ± 00 ^ns^	1
*C. albicans*	20 ± 0.015 **	20 ± 0.0001 *	1	20 ± 0.0009 *	20 ± 00 ^ns^	1
*Candida* spp.	20 ± 00 ^ns^	20 ± 00 ^ns^	1	20 ± 0.002 *	20 ± 0.007 *	1
*F. solani*	30 ± 00 ^ns^	30 ± 0.0002 *	1	30 ± 00 ^ns^	30 ± 00 ^ns^	1
*A. fischeri*	20 ± 0.014 **	20 ± 00 ^ns^	1	20 ± 00 ^ns^	20 ± 00 ^ns^	1

The experiment was performed in minimum 2 replicates with ** *p* ≤ 0.01, * *p* ≤ 0.05; ^ns^: not significant.

## Data Availability

Data are available upon request.
